# PEOPLE LIVING WITH DEMENTIA AND THEIR FAMILY MEMBERS INFORM A PERSON-CENTERED MEASURE OF “HOME TIME”

**DOI:** 10.1093/geroni/igae098.0665

**Published:** 2024-12-31

**Authors:** Nathan Boucher, Kevin McKenna, Justine Seidenfeld, Judith Vick, James Burke, Brenda Plassman, Megan Shepherd-Banigan, Courtney Van Houtven

**Affiliations:** Duke University, Durham, North Carolina, United States; Duke University School of Medicine, Durham, North Carolina, United States; Duke University Health System, Durham, North Carolina, United States; Yale University School of Medicine, New Haven, Connecticut, United States; Duke University School of Medicine, Durham, North Carolina, United States; Duke University Medical Center, Durham, North Carolina, United States; Duke University Medical Center, Durham, North Carolina, United States; Duke University School of Medicine, Durham, North Carolina, United States

## Abstract

To build a person-centered “hometime” measure for use for persons living with dementia (PLWD, inclusive of mild cognitive impairment), we conducted in-depth interviews with PLWD and spouses who support PLWD to understand quality-of-life (QOL) at home versus health facility settings. We interviewed 32 PLWD (age 59-93) and 40 spouses with recent experience in these settings: PLWD in inpatient (12 PLWD; 15 spouses), emergency (8 PLWD; 12 spouses), post-acute skilled nursing (4 PLWD; 4 spouses), outpatient surgery (PLWD 8; spouse 6), and assisted living (3 spouses). Applied thematic analysis generated four themes: 1) Emotions: Participants were frustrated by limited control in health settings. Spouses described some relief, but guilt when the PLWD was in health facility settings. PLWD described being a burden to spouses. 2) Care Challenges: Tasks, including activities of daily living, exceeding spouses’ capabilities or worsening physical/cognitive status decreased QOL at home. Spouses had fewer care duties while PLWD were in health settings. 3) Activities: Time spent with spouse, pets, television, reading, and games provided satisfaction across settings. 4) Community Involvement: Spouses had low community involvement; little changed whether PLWD was in health settings or at home. Many spouses continued to check on PLWDs’ eating, bathing, or medication administration when in a facility. These data in concert with claims data provide insights on value/quality of time at home versus health settings. Our findings inform an emerging “hometime” measure and indicate enhanced task support and social support may raise caregivers’ QOL and help avoid visits to health facilities for PLWD.

